# The Requirements of Local Anæsthesia

**Published:** 1907-04-27

**Authors:** J. Aikman

**Affiliations:** Birnam, Guernsey


### THE REQUIREMENTS OF LOCAL ANÆSTHESIA.

OK

By J. AIKMAN, M.D.Glasg., Birnam, Guernsey.

It is interesting and instructive to follow the movement which began with the introduction of general anæsthesia about the middle of the last century, which extended the domain of antiseptic surgery into new regions, and is now trending back to a localised anæsthesia produced by certain vegetable and animal alkaloids. General anæsthesia favoured the work of the handicraftsman; antiseptics opened up to him regions of the body hitherto sealed against his prowess; local anæsthesia in its perfection should free him from the dangers and responsibilities which are inseparable from the production of complete unconsciousness. Very early in the development of the antiseptic system, Lord Lister called attention to the anæsthetic effect of pure carbolic acid when applied to the wound in cases of compound fracture; he thought that the acid then tended to allay the restlessness of the patient after the first dressing. The experience of surgeons has urged that the same effect on the operator's hands, even from weak dilutions, is an argument in favour of aseptic rather than antiseptic surgery. Local anæsthesia by freezing next occupied attention, and it was noted that the numbing was more easily obtained when the part was deprived of its blood supply by a tourniquet. Later cocaine claimed favour as a local anæsthetic, but its effect on the heart was prejudicial in some cases. Here, again, the tourniquet limited the danger and accentuated the effect, but did not free the patient from certain results. Then it was recognised that the active substance of the suprarenal gland contracted the local blood-vessels, and when added to cocaine, prevented in great measure its absorption. The exact measure of the adrenalin required was a difficulty; if it was in excess it impaired the power of the tissues to repair the surgical injury; moreover, it was sometimes followed by congestion of the part and signs which might be interpreted as failing power in the phagocytes or a deficiency of the opsonins. Towards the solution of that difficulty many suggestions have been made, the most reasonable taking the form of graduated solutions enclosed in sterilised glass capsules. The requirements of the solution are obvious; it must contain enough of the local anæsthetic and not too much rena-glandin. There is a further word as to whether cocaine or eucaine is the better agent. Cocaine has the more rapid action, but the effect lasts over a short period, which may serve the needs of the surgeon, but does not maintain the restfulness of the part until this has become accustomed to its dressings—as the numbing of carbolic acid did in cases of compound fracture. In this respect eucaine acts better. One test of a good capsule is that it not only produces complete loss of sensation, but also controls weeping from scraped surfaces where such are left after the destruction of suppurating glands. That this is due to an effect of the adrenalin is evidenced by the result of dressing, the leaking surfaces tending to ulcerate on the skin enfeebled by varicose veins. The rena-glandin impregnated absorbent cotton wool forms a most useful dressing in such cases.

